# Blood Biomarkers in Moderate-To-Severe Traumatic Brain Injury: Potential Utility of a Multi-Marker Approach in Characterizing Outcome

**DOI:** 10.3389/fneur.2015.00110

**Published:** 2015-05-26

**Authors:** Alex P. Di Battista, John E. Buonora, Shawn G. Rhind, Michael G. Hutchison, Andrew J. Baker, Sandro B. Rizoli, Ramon Diaz-Arrastia, Gregory P. Mueller

**Affiliations:** ^1^Faculty of Medicine, Institute of Medical Science, University of Toronto, Toronto, ON, Canada; ^2^Defence Research and Development Canada, Toronto Research Centre, Toronto, ON, Canada; ^3^Keenan Research Centre, Li Ka Shing Knowledge Institute, St. Michael’s Hospital, Toronto, ON, Canada; ^4^Department of Anatomy, Physiology and Genetics, Uniformed Services University of the Health Sciences, Bethesda, MD, USA; ^5^US Army Graduate Program in Anesthesia Nursing, Fort Sam Houston, TX, USA; ^6^Faculty of Kinesiology and Physical Education, David L. MacIntosh Sport Medicine Clinic, University of Toronto, Toronto, ON, Canada; ^7^Department of Anesthesia, University of Toronto, Toronto, ON, Canada; ^8^Department of Surgery and Critical Care Medicine, University of Toronto, Toronto, ON, Canada; ^9^Center for Neuroscience and Regenerative Medicine, Uniformed Services University of the Health Sciences, Bethesda, MD, USA

**Keywords:** s100B, GFAP, NSE, BDNF, MCP-1, ICAM-5, PRDX-6

## Abstract

**Background:**

Blood biomarkers are valuable tools for elucidating complex cellular and molecular mechanisms underlying traumatic brain injury (TBI). Profiling distinct classes of biomarkers could aid in the identification and characterization of initial injury and secondary pathological processes. This study characterized the prognostic performance of a recently developed multi-marker panel of circulating biomarkers that reflect specific pathogenic mechanisms including neuroinflammation, oxidative damage, and neuroregeneration, in moderate-to-severe TBI patients.

**Materials and methods:**

Peripheral blood was drawn from 85 isolated TBI patients (*n* = 60 severe, *n* = 25 moderate) at hospital admission, 6-, 12-, and 24-h post-injury. Mortality and neurological outcome were assessed using the extended Glasgow Outcome Scale. A multiplex platform was designed on MULTI-SPOT^®^ plates to simultaneously analyze human plasma levels of s100 calcium binding protein beta (s100B), glial fibrillary acidic protein (GFAP), neuron specific enolase (NSE), brain-derived neurotrophic factor (BDNF), monocyte chemoattractant protein (MCP)-1, intercellular adhesion molecule (ICAM)-5, and peroxiredoxin (PRDX)-6. Multivariable logistic regression and area under the receiver-operating characteristic curve (AUC) were used to evaluate both individual and combined predictive abilities of these markers for 6-month neurological outcome and mortality after TBI.

**Results:**

Unfavorable neurological outcome was associated with elevations in s100B, GFAP, and MCP-1. Mortality was related to differences in six of the seven markers analyzed. Combined admission concentrations of s100B, GFAP, and MCP-1 were able to discriminate favorable versus unfavorable outcome (AUC = 0.83), and survival versus death (AUC = 0.87), although not significantly better than s100B alone (AUC = 0.82 and 0.86, respectively).

**Conclusion:**

The multi-marker panel of TBI-related biomarkers performed well in discriminating unfavorable and favorable outcomes in the acute period after moderate-to-severe TBI. However, the combination of these biomarkers did not outperform s100B alone.

## Introduction

The multifactorial nature of secondary injury after traumatic brain injury (TBI), especially the complex networks of molecular pathways mediating cellular damage in different brain regions, has confounded attempts to elucidate the pathology underlying injury progression (1). In addition, the extracranial effects of trauma must be considered as these are often critical factors in the death of TBI patients (2). In recent years, the application of brain-specific markers of neuronal, glial, and axonal damage, identified in the peripheral blood, has shown potential clinical utility in neurointensive care as diagnostic, prognostic, and monitoring adjuncts (3). Identifying sensitive and reliable biomarkers associated with patient outcome may improve our understanding of structural brain damage or underlying cellular pathogenesis and regenerative mechanisms after brain trauma (4). This information can be used to guide future basic and clinical research, with the aim of improving patient care and outcomes (5).

The peripheral blood can be used as a source of biomarkers indicative of neuropathology across the spectrum of mild to severe brain injury (6). It has been proposed that damage to brain tissue after a TBI may initiate local metabolic and inflammatory processes, resulting in the release of a number of inflammatory mediators and damage-associated molecular pattern (DAMP) molecules (7, 8). These molecules then cross a dysfunctional blood brain barrier (BBB) and enter the circulation to recruit peripheral immune cells to the brain, initiating bidirectional communication between the CNS and the systemic immune system (8–10). Thus, peripheral blood samples may contain molecules derived from the CNS and/or the periphery as a secondary response to injury.

Several blood-borne biomarker candidates have been investigated either individually or collectively across the spectrum of TBI severity. In particular, s100 calcium binding protein beta (s100B) and neuron specific enolase (NSE) have been widely studied, and elevated circulating concentrations of these markers may be associated with secondary injury progression (11) and poor prognosis (12, 13). Additionally, inflammatory cytokines, such as Interleukin (IL)-1β, -6, -8, and -10, are associated with poor outcome following severe TBI (14, 15). However, examination of single DAMP molecules, such as s100B, may lack specificity due to extracranial sources (16, 17). Furthermore, systemic inflammatory markers are often pleiotropic in nature and thus difficult to individually link to TBI.

Thus, a multi-marker approach to characterizing TBI outcome has been advocated, since the simultaneous estimation of multiple markers to establish a “biological signature” may prove more effective in encompassing the multisystemic character of secondary injury pathology, and may increase diagnostic and prognostic accuracy (18). For example, Gradisek et al. (19) demonstrated that admission blood levels of s100B and glial fibrillary acidic protein (GFAP) together accurately discerned survivors from non-survivors 1 year following TBI, and Diaz-Arrastia et al. (20) found the combination of ubiquitin C-terminal hydrolase-1 (UCH-L1) and GFAP out performed either marker individually in discriminating TBI patients from healthy controls. However, these studies did not include markers reflecting additional secondary injury processes such as inflammation and oxidative damage. In view of this, Buonora et al. (21) recently employed a multivariate approach to TBI diagnosis by simultaneously assessing seven blood biomarkers, each associated with a specific TBI-related injury process: NSE relating to neuronal injury; brain-derived neurotrophic factor (BDNF) for neuronal repair; peroxiredoxin (PRDX)-6 for oxidative damage; GFAP and s100B for glial damage; monocyte chemoattractant protein (MCP)-1 for immune activation; intercellular adhesion molecule (ICAM)-5 for disruption of intercellular adhesion processes. The authors reported that a novel multi-marker panel, detectable in peripheral blood, demonstrated diagnostic utility specifically for mTBI and potentially for the full spectrum of brain injury (21). However, while this multi-marker panel shows promise in acute TBI diagnosis, the prognostic utility of these markers for longer-term outcome in more severely injured patients has not been assessed.

Hence, the purpose of this study was to further examine a recently developed panel of 7 biomarkers in a cohort of moderate-to-severe TBI patients. The two specific aims were (1) to characterize the temporal profile of plasma s100B, GFAP, NSE, BDNF, MCP-1, ICAM-5, and PRDX-6 concentrations at four time-points within the first 24 h of hospital admission, stratified according to patient outcomes, (2) to compare the individual and collective utility of these markers in discriminating between favorable and unfavorable patient outcomes.

## Materials and Methods

### Study population and enrollment

Potential study participants were admitted to Sunnybrook Health Sciences Centre and St. Michael’s Hospital (Toronto, ON, Canada). Upon admission, the trauma team/emergency room personnel enrolled patients who met the initial criteria of sustaining an isolated TBI, defined by a Glasgow Coma Score of <13 and a non-head abbreviated injury score (AIS) ≤2; TBI patients were further dichotomized into moderate (GCS 9–12) and severe (GCS 3–8) injury. Consent for enrollment was obtained from a substitute decision maker. If this was not possible, consent was delayed in accordance with the Tri-Council Policy Agreement for Research in Emergency Health Situations (Article 2.8), and obtained from next-of-kin. If the patient recovered sufficiently to provide consent, their consent was also obtained. Patients were excluded in the following cases: an elapsed time between trauma and hospital admission in excess of 3 h, <16 years of age, pregnant, lacking vital signs prior to admission, or clinically brain dead on admission. The study protocol was approved by the Research Ethics Boards at Sunnybrook Health Sciences Centre and St. Michaels Hospital. Blood samples were drawn from healthy volunteers after obtaining written informed consent in accordance with the principles of the Declaration of Helsinki.

### Study design and procedures

At hospital admission, demographic data were obtained from study patients along with a number of clinical indices. This process has been described in detail previously (21). Briefly, mechanism of injury, elapsed time from trauma to the emergency room, and neurological status were recorded. Past medical history was obtained along with routine laboratory exams, including a computerized tomography (CT) scan. All significant events up to hospital discharge, death or 28 days were recorded. In the instance of patient death, cause of death was recorded as TBI or non-TBI related. The extended Glasgow Outcome Scale (GOSE) was used to assess patient outcome at hospital discharge, at 28 days, and at 6-month post-injury.

### Blood sample collection and analysis

Venous blood samples were collected at hospital admission, and then again at 6-, 12-, and 24-h post-injury. Samples were drawn into 10-ml K_2_EDTA (with 4 mM sodium metabisulfite [Na_2_S_2_O_5_]) or 10-ml sodium heparin vacutainers (Vacutainer, Becton Dickinson, Rutherford, NJ, USA). The samples were immediately centrifuged at 1600 × *g* for 15 min at 4°C, and the plasma supernatant was aliquoted into six (1–2 ml) aliquots and frozen at −70°C until subsequent analysis. Analysis of BDNF, GFAP, MCP-1, ICAM-5, NSE, and s100B plasma concentrations was performed using a multiplex immunoassay system, while plasma PRDX-6 was assessed using a single-plex immunoassay system. All assays were performed on the meso scale discovery (MSD) SECTOR^®^ Imager 6000 (MSD, Gainsburg, MD, USA), as previously described (21). The lower limit of detection (LLOD) and lower limit of quantitation (LLOQ) were defined as 3 and 10 times the SD of the averaged 0 for each assay, respectively. All samples were run in triplicate- <3 and <10% intra- and inter-assay variability, respectively, was observed for all multiplex samples, while all single-plex samples displayed <4 and <13% intra- and inter-assay variability, respectively.

### Statistical analysis

Clinical and demographic data are represented as the mean ± SD unless otherwise noted. The normality of each variable was assessed before the appropriate statistical test was applied. To examine 6-month neurological outcome, patients were dichotomized into favorable (GOSE 5–8) and unfavorable (GOSE 1–4) outcome groups. Similarly, patients were also stratified into two groups, “Lived” and “Died” to assess mortality. Group classifications for both neurological outcome and mortality were analyzed at each time-point using either a Student’s *t*-test or Mann–Whitney *U*, where appropriate. To investigate the prognostic utility of peripheral blood markers, admission levels of each marker were used to discriminate between favorable versus unfavorable 6-month neurological outcome, and lived versus died using single or multi-marker receiver–operator characteristic (ROC) curves. Multiple-marker ROC curves were compared to single-marker curves using binary logistic regression analysis followed by the *chi-squared* statistic. Statistical significance was set at (*p* ≤ 0.05) for all analyses. All data were analyzed using GraphPad Prism Version 6.0d (GraphPad Inc., CA, USA) and Stata Version 13.1 (StataCorp, TX, USA).

## Results

### Demographic and clinical characteristics

Clinical and demographic data for all TBI patients are described in Table 1. Of the 85 TBI patients, 25 were moderate, and 60 severe. The average age of the patients was 45.8 ± 21.9 years. Twenty patients (23.5%) developed sepsis, and 50 patients (58.8%) had an unfavorable neurological outcome as described by a 6-month GOSE score of 1–4. The mortality rate was 28.2%, and half of all deaths resulted from organ failure.

**Table 1:** **Demographic, clinical, and outcome data of brain-injured patients**.

Characteristics	All patients (*n* = 85)	Moderate TBI (*n* = 25)	Severe TBI (*n* = 60)
**Demographics**
Age (years)	45.8 ± 21.9	47.9 ± 21.7	44.9 ± 22.2
Male gender – *n* (%)	66 (77.6)	19 (76.0)	47 (78.3)
**Clinical characteristics**
Trauma type – *n* (%)			
Blunt	83 (97.6)	24 (96.0)	59 (98.3)
Penetrating	2 (2.3)	1 (4.0)	1 (1.7)
Time to ED (min)	79.6 ± 56.4	72.3 ± 52.0	82.7 ± 58.2
ISS score	23.6 ± 11.0	19.1 ± 12.5	25.4 ± 9.8
AIS head	4.2 ± 1.1	3.6 ± 1.2	4.4 ± 1.0
GCS	6.5 ± 3.3	10.8 ± 1.2	4.63 ± 2.0
Positive CT – *n* (%)	70 (82.3)	18 (72.0)	52 (86.7)
Positive serum ethanol – *n* (%)	27 (31.8)	10 (40.0)	17 (31.7)
Pre-injury comorbidities – *n* (%)	27 (31.8)	11 (44.0)	16 (26.7)
Pre-injury beta-blocker use – *n* (%)	4 (4.7)	1 (4.0)	3 (5.0)
Neurosurgical intervention – *n* (%)	25 (29.4)	3 (12.0)	22 (36.7)
**Outcomes**
Mortality *– n* (%)	24 (28.2)	–	24 (40.0)
Unfavorable outcome – *n* (%)	50 (58.8)	8 (32.0)	42 (70.0)
Sepsis infection – *n* (%)	20 (23.5)	6 (24.0)	14 (23.3)
Organ failure – *n* (%)	12 (14.1)	–	12 (20.0)

*TBI, traumatic brain injury; ED, emergency department; ISS, injury severity score; AIS, abbreviated injury scale; GCS, Glasgow coma scale; GOSE, Glasgow outcome scale extended*.

*Moderate TBI = GCS 9–12; Severe TBI = GCS 3–8*.

*Unless otherwise stated, results are expressed as mean ± SD*.

### Plasma concentrations of neuroinjury markers stratified by clinical indices

#### 6-Month Neurological Outcome

**Figure 1:**
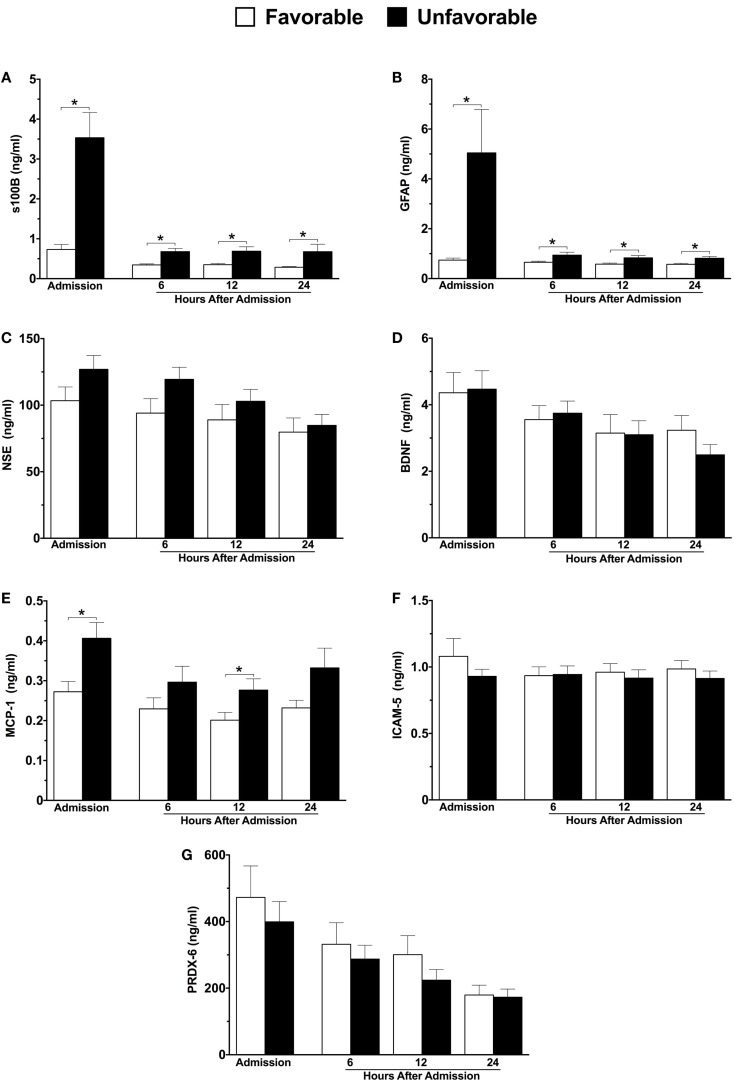
**Plasma concentrations of neuroinjury biomarkers in combined moderate-to-severe TBI patients within the first 24 h of hospital admission, stratified according to 6-month neurological outcome using the extended Glasgow outcome scale (GOSE) score**. Favorable outcome = GOSE 5–8, *n* = 35; Unfavorable outcome = GOSE 1–4, *n* = 50. s100 calcium binding protein **(A)**, glial fibrillary acidic protein **(B)**, neuron specific enolase **(C)**, brain-derived neurotrophic factor **(D)**, monocyte chemoattractant protein-1 **(E)**, intercellular adhesion molecule-5 **(F)**, peroxiredoxin-6 **(G)**. Sample sizes may vary. *p ≤ 0.05 by Student’s *t-*test or Mann–Whitney *U*, where appropriate, versus patients with a favorable outcome.

Significant differences in three of the seven markers were identified in patients with unfavorable versus favorable 6-month neurological outcome (Figures 1A–G). The largest difference was observed in GFAP, which displayed a nearly seven-fold elevation in patients with unfavorable outcome at hospital admission versus those with a favorable outcome (5.05 versus 0.74 ng/ml), and remained significantly elevated at each sampled time-point within the first 24 h (Figure 1B). Admission plasma s100B levels displayed a near four-fold increase in those with unfavorable outcome (Figure 1A). Similar to GFAP, s100B remained significantly elevated throughout the first 24 h (Figure 1A). Admission and 12 h plasma concentrations of MCP-1 were also significantly elevated in patients with an unfavorable outcome (Figure 1E).

#### Mortality

**Figure 2:**
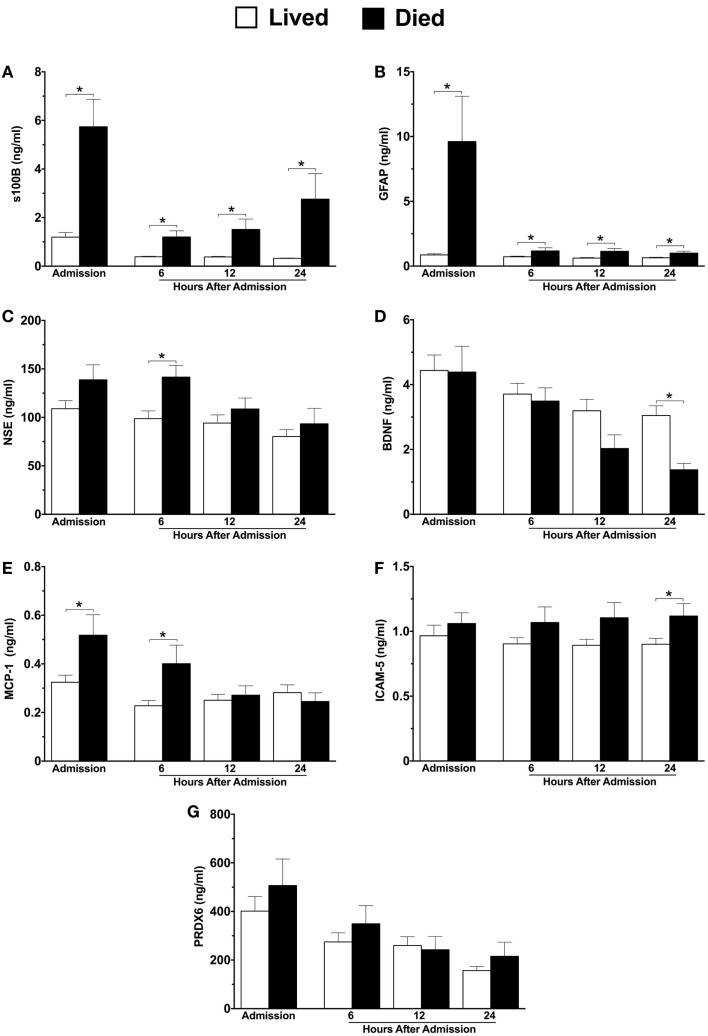
**Plasma concentrations of neuroinjury biomarkers in combined moderate-to-severe TBI patients within the first 24 h of hospital admission, stratified by patients who lived versus died**. Lived, *n* = 61; Died, *n* = 24. s100 calcium binding protein **(A)**, glial fibrillary acidic protein **(B)**, neuron specific enolase **(C)**, brain-derived neurotrophic factor **(D)**, monocyte chemoattractant protein-1 **(E)**, intercellular adhesion molecule-5 **(F)**, peroxiredoxin-6 **(G)**. Sample sizes may vary. *p ≤ 0.05 by Student’s *t-*test or Mann–Whitney *U*, where appropriate, versus patients who survived.

Six of the seven neuroinjury markers were significantly different between survivors and non-survivors (Figures 2A–G). Similar to 6-month neurological outcome, GFAP levels at hospital admission were significantly elevated among patients who died compared with those who lived (eleven-fold increase, 9.612 versus 0.86 ng/ml) (Figure 2B). GFAP levels remained significantly elevated in patients who died at all sampled time-points (Figure 2B). Admission levels of s100B were nearly five-fold higher in patients who died as compared to those who lived, and remained elevated at all time-points (Figure 2A). At 6 h, both NSE and MCP-1 levels were significantly higher in non-survivors versus survivors (Figures 2C,E, respectively). At 24 h, BDNF and ICAM-5 levels were significantly decreased and increased, respectively, in patients who died (Figures 2D,F, respectively).

### ROC curve analysis

See Table 2 for individual marker AUC values – each marker was evaluated for their ability to discriminate favorable versus unfavorable 6-month neurological outcome, and survival versus death. Admission plasma concentrations of s100B, GFAP, and MCP-1 were significantly associated with both unfavorable outcome and mortality (Table 2). s100B displayed the strongest relationship to adverse 6-month neurological outcome (AUC = 82.5) and mortality (AUC = 86.5) (Table 2).

**Table 2:** **Single-marker AUC values for clinical indices**.

Marker	Unfavorable outcome	Mortality
s100B	82.5 ± 0.05*	86.5 ± 0.04*
GFAP	71.5 ± 0.06*	79.5 ± 0.06*
NSE	59.5 ± 0.06	61.3 ± 0.07
BDNF	48.5 ± 0.07	47.8 ± 0.08
MCP-1	65.3 ± 0.06*	70.2 ± 0.06*
ICAM-5	46.4 ± 0.07	62.4 ± 0.07*
PRDX-6	48.6 ± 0.07	56.1 ± 0.07

*Data represented as AUC ± SE*.

*p < 0.05 by logistic regression analysis.

**Figure 3:**
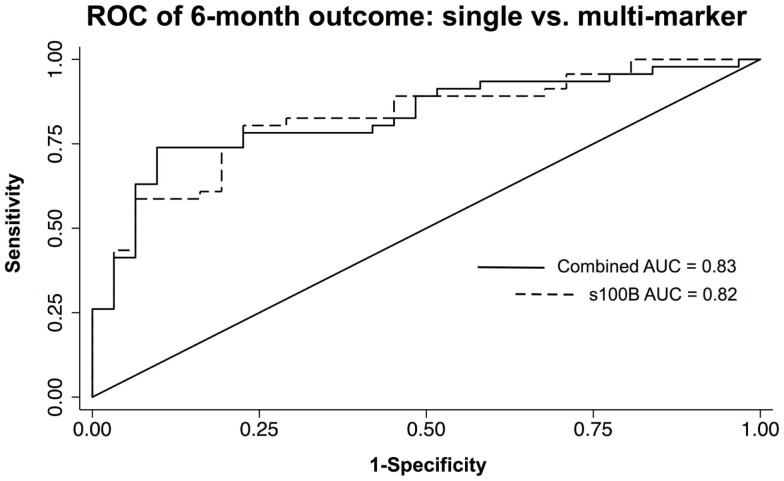
**ROC curves of neuroinjury biomarkers used to discriminate unfavorable versus favorable 6-month neurological outcome in TBI patients**. A combined AUC consisting of s100B, GFAP, and MCP-1 was not statistically better than s100B alone in discriminating unfavorable from favorable outcome by *chi-squared*.

**Figure 4:**
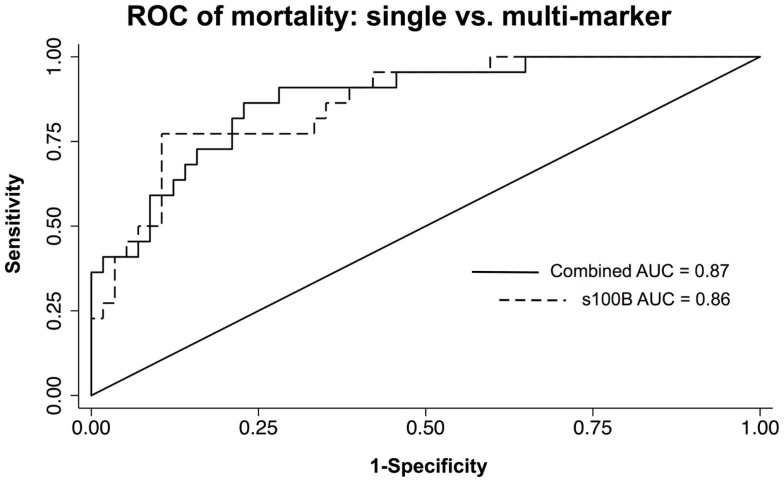
**ROC curves of neuroinjury markers used to discriminate death from survival in TBI patients**. A combined AUC consisting of s100B, GFAP, and MCP-1 was not statistically better than s100B alone in discriminating death from survival by *chi-squared*.

Multivariate models were created using the significant individual predictors of unfavorable 6-month neurological outcome and mortality to create multi-marker ROC curves. This model, which consisted of s100B, GFAP, and MCP-1, did not significantly differ from s100B alone in discriminating favorable from unfavorable outcome (AUC = 0.83 versus AUC = 0.82) (Figure 3). Similarly, multivariate model discrimination of mortality from survival (AUC = 0.87) was not significantly different than that of s100B alone (AUC = 0.86) (Figure 4).

## Discussion

This study characterized peripheral blood s100B, GFAP, NSE, BDNF, MCP-1, ICAM-5, and PRDX-6 levels over the first 24 h post-hospital admission in 85 moderate-to-severe TBI patients. We identified significantly elevated concentrations of s100B and GFAP at all sample time points (admission, 6-, 12-, 24-h) in patients with unfavorable neurological outcome at 6 months and in those who died. This is in agreement with previous studies that found high levels of s100B and GFAP were associated with poor outcome at 1–6 months post-TBI. While we found no relationship between NSE and 6-month neurological outcome, mortality was associated with elevations of NSE at 6 h after hospital admission. Our results are in accord with prior findings that identified a relationship between blood NSE levels early after injury, and mortality (19). Furthermore, our results are also consistent with others who found elevated NSE levels beyond the first 24 h after hospital admission in association with patient death (22, 23). However, previous studies have also reported a correlation between NSE and unfavorable neurological outcome (22–26). The reasons for these discordant findings are unclear, but may relate to methodological differences involving sample times (22), heterogeneous patient populations (23), and varied outcome stratification measures (24).

Both CNS and systemic inflammation are critical components to secondary injury after TBI (9, 27, 28). While the role of innate immunity after TBI is poorly understood, it has been theorized that local inflammation, in response to the initial trauma initiates a cascade of events that include the recruitment of peripheral leukocytes to the brain, potentially exacerbating brain tissue injury (29, 30). In view of this, ICAM-5 and MCP-1 are inflammatory molecules potentially involved in aiding peripheral leukocyte mobilization. ICAM-5 is found primarily in telencephalic neurons (31), and is involved in the activation and migration of leukocytes across the endothelium (32). MCP-1 is a chemoattractant protein secreted from both mononuclear and neuronal cells that facilitates monocyte and macrophage mobilization (29, 33, 34). In the present study, ICAM-5 levels were significantly elevated at 24 h after hospital admission in non-survivors compared to survivors. Our previous work characterized peripheral blood ICAM-5 concentrations after TBI, and identified decreased levels in moderate and severe TBI patients acutely after hospital admission (21). To our knowledge, no studies have examined the association between plasma ICAM-5 levels and patient outcome in the acute period after TBI. It is possible that our previously reported decrease of ICAM-5 acutely after moderate and severe TBI may have masked elevations observed in a subset of non-survivors. Additionally, we found MCP-1 was significantly increased at admission and 12 h in those with unfavorable neurological outcome, as well as at admission and 6 h in those who died. These findings are aligned with previous work by Rhodes et al. (35), who identified elevated MCP-1 levels in the blood of severe TBI patients who died compared with survivors. Furthermore, in a rodent model of TBI, Semple et al. (29) identified a role for MCP-1 in delaying functional recovery after closed head injury. In the present study, our findings in both ICAM-5 and MCP-1 are consistent with immunopathological communication between the CNS and the periphery.

Secondary injury after TBI not only encompasses damage-related processes that worsen brain injury, but also an altered state of tissue regeneration and repair (36). In view of this, BDNF has been proposed as a potential biomarker reflecting the neuroregenerative response to TBI (37). BDNF is the most abundant brain neurotrophin, involved in promoting neuron survival, differentiation, and outgrowth (38, 39). In the present study, we found significantly decreased plasma levels of BDNF at 24 h after hospital admission in non-survivors versus survivors. In our previous work, we identified elevated levels of circulating BDNF within 24 h of hospital admission in moderate and severe TBI patients (21). This suggests that BDNF may be upregulated after injury in response to neuronal damage, although an inadequate BDNF response may negatively impact patient outcome. In view of this, animal model research has identified associations between increased hippocampal BDNF levels and functional recovery after TBI (40), and the Val66Met polymorphism of the BDNF gene, which interferes with BDNF secretion, has been associated with poor outcome in human subarachnoid hemorrhage survivors (41). However, this polymorphism has also been associated with improved functional recovery of executive functioning after TBI (42). It is important to note that these studies evaluated BDNF gene alleles in association with patient recovery from months to years after injury, while we assessed plasma BDNF protein concentrations in the acute period after TBI. Differences in both study design and experimental approach may account for the heterogeneity in findings. We also characterized PRDX-6, an antioxidant enzyme found primarily in astrocytes (43). PRDX-6 has a purported role in protecting brain tissue from neurodegeneration, specifically preventing neuronal damage via lipid peroxidation and cell death signaling related to hypoxia (43, 44). However, the role of PRDX-6 in TBI is unclear. Manevich et al. (43) found elevated levels of the oxidated (inactive) form of PRDX-6 in the CSF of severe TBI patients, and we previously found elevated plasma levels of PRDX-6 in TBI patients (21). However, in the present study, we did not find any association between plasma levels of PRDX-6 and unfavorable neurological outcome or mortality. It is possible that PRDX-6 has utility as a diagnostic marker in TBI, while its prognostic capabilities may be limited.

In the current study, a multiple-marker model consisting of s100B, GFAP, and MCP-1 did not yield significantly higher AUC values for discriminating unfavorable from favorable outcomes after TBI compared to s100B alone. Previous TBI studies that have characterized biomarkers in relation to patient outcomes have often evaluated markers individually (14, 24), or used a combined-approach consisting of a single marker with other clinical indices (e.g., CT scan results) (3, 22). However, the purpose of our study was to evaluate single versus multiple biomarkers alone, absent clinical indices, and few studies have done this to date. In agreement with our findings, DeFazio et al. (45) found that admission levels of plasma s100B were associated with poor status in severe TBI patients at 72 h, and that this relationship was not improved upon by a multivariate model including D-dimer and s100B at 24 h. Conversely, Berger et al. (46) found the combination of s100B, NSE, and myelin basic protein (MBP) improved outcome prediction in pediatric TBI. Although we did not find a significant difference in outcome discrimination between multiple and individual markers, we did not assess MBP, and only evaluated adult TBI patients. In addition, Gradisek et al. (19) found s100B together with GFAP improved 1-year mortality prediction. However, these markers were used to predict mortality in patients who succumbed to brain-related deaths. In the same study, there was no association between these biomarkers and non-brain-related deaths such as multiple organ failure (19). In view of this, we included all causes of mortality in our non-survivor group. It is likely that following a TBI, brain-related death and subsequent organ failure reflect different pathological mechanisms, and may require specific biological signatures for their detection. Furthermore, our finding of s100B alone to be associated with global outcome and mortality following TBI may reflect its role as an inflammatory mediator in the peripheral blood. Beyond its role as a possible marker of brain tissue damage, s100B also acts as a ligand on numerous cell types, including peripheral leukocytes, which express the receptor for advanced glycation end-products (RAGE) (47, 48). This signaling cascade propagates an inflammatory response (47) and thus may play a role in post-TBI complications such as multiple organ failure, which is known to have an inflammatory pathology (49).

Despite having a robust sample size to characterize TBI, a larger patient cohort would have been required for further sub-stratifications, particularly to dichotomize moderate and severe patients; we were unable to assess outcome and mortality in isolated severe and moderate injures due to a lack of moderately injured patients with unfavorable outcomes. Future studies should also consider incorporating multiple-marker panels relating biological molecules to brain-specific clinical indices such as intracranial pressure and specific CT scan classifications, as well as associated systemic outcomes of TBI including sepsis and multiple organ failure.

## Conclusion

Traumatic brain injury elicits the release of a number of neuroinjury molecules that are easily assessed in the peripheral blood and hold promise in the clinical management of patients. In the present study, elevated levels of s100B, GFAP, and MCP-1 in the peripheral blood of TBI patients within 24 h of hospital admission were associated with both unfavorable 6-month neurological outcome and death. Despite promising evidence of multi-marker algorithms displaying enhanced utility to discriminate between healthy individuals and TBI patients, under the current approach, the combination of each individual marker did not yield greater integrated discrimination beyond the single-marker s100B in moderate-to-severe TBI.

## Author Contributions

All authors were involved in the drafting and revising of the manuscript, as well as the interpretation of the data. JB, GM, RD-A, SR, AB, and SR all contributed substantially to the conception and design of this work. AD, MH, JB, GM, and SR contributed substantially to the acquisition and analysis of the data.

## Conflict of Interest Statement

The authors declare that the research was conducted in the absence of any commercial or financial relationships that could be construed as a potential conflict of interest.

## References

[1] McKee AC Daneshvar DH The neuropathology of traumatic brain injury. Handb Clin Neurol (2015) 127:45–66. 10.1016/B978-0-444-52892-6.00004-0 25702209 PMC4694720

[2] Gaddam SSK Buell T Robertson CS Systemic manifestations of traumatic brain injury. Handb Clin Neurol (2015) 127:205–18. 10.1016/B978-0-444-52892-6.00014-3 25702219

[3] Papa L Robertson CS Wang KKW Brophy GM Hannay HJ Heaton S Biomarkers improve clinical outcome predictors of mortality following non-penetrating severe traumatic brain injury. Neurocrit Care (2015) 22:52–64. 10.1007/s12028-014-0028-2 25052159

[4] Mondello S Hayes RL. Biomarkers. Handb Clin Neurol (2015) 127:245–65. 10.1016/B978-0-444-52892-6.00016-7 25702221

[5] Wang KKW Moghieb A Yang Z Zhang Z Systems biomarkers as acute diagnostics and chronic monitoring tools for traumatic brain injury. In: Proc. SPIE 8723, Sensing Technologies for Global Health, Military Medicine, and Environmental Monitoring III, Vol. 87230O (2013). 10.1117/12.2020030

[6] Di Battista AP Rhind SG Baker AJ. Application of blood-based biomarkers in human mild traumatic brain injury. Front Neurol (2013) 4:44. 10.3389/fneur.2013.00044 23641234 PMC3640204

[7] Balu R. Inflammation and immune system activation after traumatic brain injury. Curr Neurol Neurosci Rep (2014) 14:484–484. 10.1007/s11910-014-0484-2 25138025

[8] Finnie JW Pathology of traumatic brain injury. Vet Res Commun (2014) 38:297–305. 10.1007/s11259-014-9616-z 25178417

[9] Hinson HE Rowell S Schreiber M. Clinical evidence of inflammation driving secondary brain injury: a systematic review. J Trauma Acute Care Surg (2015) 78:184–91. 10.1097/TA.0000000000000468 25539220 PMC4297199

[10] Denes A Miyan JA Brain-immune interactions in health and disease. Front Neurosci (2014) 8:382 10.3389/fnins.2014.00382 25520609 PMC4253740

[11] Wolf H Frantal S Pajenda G Leitgeb J Sarahrudi K Hajdu S. Analysis of s100 calcium binding protein B serum levels in different types of traumatic intracranial lesions. J Neurotrauma (2015) 32:23–7. 10.1089/neu.2013.3202 25068442

[12] Lesko MM O’Brien SJ Childs C Bouamra O Rainey T Lecky F. Comparison of several prognostic tools in traumatic brain injury including S100B. Brain Inj (2014) 28:987–94. 10.3109/02699052.2014.890743 24655224

[13] Egea-Guerrero JJ Revuelto-Rey J Murillo-Cabezas F Muñoz-Sánchez MA Vilches-Arenas A Sánchez-Linares P Accuracy of the S100 βprotein as a marker of brain damage in traumatic brain injury. Brain Inj (2012) 26:76–82. 10.3109/02699052.2011.635360 22149446

[14] Ferreira LCB Regner A Miotto KDL Moura SD Ikuta N Vargas AE Increased levels of interleukin-6, -8 and -10 are associated with fatal outcome following severe traumatic brain injury. Brain Inj (2014) 28:1311–6. 10.3109/02699052.2014.916818 24830571

[15] Chiaretti A Genovese O Aloe L Antonelli A Piastra M Polidori G Interleukin 1β and interleukin 6 relationship with paediatric head trauma severity and outcome. Childs Nerv Syst (2005) 21:185–93. 10.1007/s00381-004-1032-1 15455248

[16] Savola O Pyhtinen J Leino TK Siitonen S Niemelä O Hillbom M. Effects of head and extracranial injuries on serum protein S100B levels in trauma patients. J Trauma (2004) 56:1229–34 discussion 1234. 10.1097/01.TA.0000096644.08735.72 15211130

[17] Undén J Bellner J Eneroth M Alling C Ingebrigtsen T Romner B. Raised serum S100B levels after acute bone fractures without cerebral injury. J Trauma (2005) 58:59–61. 10.1097/01.TA.0000130613.35877.75 15674151

[18] Yokobori S Hosein K Burks S Sharma I Gajavelli S Bullock R. Biomarkers for the clinical differential diagnosis in traumatic brain injury – a systematic review. CNS Neurosci Ther (2013) 19(8):556–65. 10.1111/cns.12127 23710877 PMC6493562

[19] Gradisek P Osredkar J Korsic M Kremzar B. Multiple indicators model of long-term mortality in traumatic brain injury. Brain Inj (2012) 26:1472–81. 10.3109/02699052.2012.694567 22721420

[20] Diaz-Arrastia R Wang KKW Papa L Sorani MD Yue JK Puccio AM Acute biomarkers of traumatic brain injury: relationship between plasma levels of ubiquitin C-terminal hydrolase-L1 and glial fibrillary acidic protein. J Neurotrauma (2014) 31:19–25. 10.1089/neu.2013.3040 23865516 PMC3880090

[21] Buonora JE Yarnell AM Lazarus R Multivariate analysis of traumatic brain injury: development of an assessment score. Front Neurol (2015) 6:68 10.3389/fneur.2015.00068/abstract 25870583 PMC4378282

[22] Olivecrona Z Bobinski L Koskinen L-OD. Association of ICP, CPP, CT findings and S-100B and NSE in severe traumatic head injury. Prognostic value of the biomarkers. Brain Inj (2015) 29:446–54. 10.3109/02699052.2014.989403 25518864

[23] Chabok SY Moghadam AD Saneei Z Amlashi FG Leili EK Amiri ZM. Neuron-specific enolase and S100BB as outcome predictors in severe diffuse axonal injury. J Trauma Acute Care Surg (2012) 72:1654–7. 10.1097/TA.0b013e318246887e 22695436

[24] Meric E Gunduz A Turedi S Cakir E Yandi M. The prognostic value of neuron-specific enolase in head trauma patients. J Emerg Med (2010) 38:297–301. 10.1016/j.jemermed.2007.11.032 18499387

[25] Žurek J Fedora M. The usefulness of S100B, NSE, GFAP, NF-H, secretagogin and Hsp70 as a predictive biomarker of outcome in children with traumatic brain injury. Acta Neurochir (Wien) (2012) 154:93–103. 10.1007/s00701-011-1175-2 21976236

[26] Vos PE Lamers KJB Hendriks JCM van Haaren M Beems T Zimmerman C Glial and neuronal proteins in serum predict outcome after severe traumatic brain injury. Neurology (2004) 62:1303–10. 10.1212/01.WNL.0000120550.00643.DC 15111666

[27] Bigler ED Neuroinflammation and the dynamic lesion in traumatic brain injury. Brain (2013) 136:9–11. 10.1093/brain/aws342 23365089

[28] Jaerve A Müller HW. Chemokines in CNS injury and repair. Cell Tissue Res (2012) 349:229–48. 10.1007/s00441-012-1427-3 22700007

[29] Semple BD Bye N Rancan M Ziebell JM Morganti-Kossmann MC. Role of CCL2 (MCP-1) in traumatic brain injury (TBI): evidence from severe TBI patients and CCL2-/- mice. J Cereb Blood Flow Metab (2010) 30:769–82. 10.1038/jcbfm.2009.262 20029451 PMC2949175

[30] Finnie JW. Neuroinflammation: beneficial and detrimental effects after traumatic brain injury. Inflammopharmacology (2013) 21(4):309–20. 10.1007/s10787-012-0164-2 23296919

[31] Yang H. Structure, expression, and function of ICAM-5. Comp Funct Genomics (2012) 2012:368938. 10.1155/2012/368938 22312318 PMC3270525

[32] Guo H Tong N Turner T Epstein LG McDermott MP Kilgannon P Release of the neuronal glycoprotein ICAM-5 in serum after hypoxic-ischemic injury. Ann Neurol (2000) 48:590–602. 10.1002/1531-8249(200010)48:4<590:AID-ANA5>3.0.CO;2-7 11026442

[33] Rhodes JKJ Sharkey J Andrews PJD. The temporal expression, cellular localization, and inhibition of the chemokines MIP-2 and MCP-1 after traumatic brain injury in the rat. J Neurotrauma (2009) 26:507–25. 10.1089/neu.2008.0686 19210118

[34] Leonard EJ Yoshimura T. Human monocyte chemoattractant protein-1 (MCP-1). Immunol Today (1990) 11:97–101. 10.1016/0167-5699(90)90035-8 2186747

[35] Rhodes J Sharkey J Andrews P. Serum IL-8 and MCP-1 concentration do not identify patients with enlarging contusions after traumatic brain injury. J Trauma (2009) 66:1591–8. 10.1097/TA.0b013e31819a0344 19509619

[36] Corps KN Roth TL McGavern DB Inflammation and neuroprotection in traumatic brain injury. JAMA Neurol (2015) 72(3):355–62. 10.1001/jamaneurol.2014.3558 25599342 PMC5001842

[37] Kaplan GB Vasterling JJ Vedak PC. Brain-derived neurotrophic factor in traumatic brain injury, post-traumatic stress disorder, and their comorbid conditions: role in pathogenesis and treatment. Behav Pharmacol (2010) 21:427–37. 10.1097/FBP.0b013e32833d8bc9 20679891

[38] Poo MM Neurotrophins as synaptic modulators. Nat Rev Neurosci (2001) 2:24–32. 10.1038/35049004 11253356

[39] Huang EJ Reichardt LF. Neurotrophins: roles in neuronal development and function. Annu Rev Neurosci (2001) 24:677–736. 10.1146/annurev.neuro.24.1.677 11520916 PMC2758233

[40] Mahmood A Goussev A Kazmi H Qu C Lu D Chopp M. Long-term benefits after treatment of traumatic brain injury with simvastatin in rats. Neurosurgery (2009) 65:187–91. discussion 191–2, 10.1227/01.NEU.0000343540.24780.D6 19574841 PMC2741617

[41] Siironen J Juvela S Kanarek K Vilkki J Hernesniemi J Lappalainen J. The Met allele of the BDNF Val66Met polymorphism predicts poor outcome among survivors of aneurysmal subarachnoid hemorrhage. Stroke (2007) 38:2858–60. 10.1161/STROKEAHA.107.485441 17761923

[42] Krueger F Pardini M Huey ED Raymont V Solomon J Lipsky RH The role of the Met66 brain-derived neurotrophic factor allele in the recovery of executive functioning after combat-related traumatic brain injury. J Neurosci (2011) 31:598–606. 10.1523/JNEUROSCI.1399-10.2011 21228168 PMC3195417

[43] Manevich Y Hutchens S Halushka PV Tew KD Townsend DM Jauch EC Peroxiredoxin VI oxidation in cerebrospinal fluid correlates with traumatic brain injury outcome. Free Radic Biol Med (2014) 72:210–21. 10.1016/j.freeradbiomed.2014.04.002 24726861 PMC4088265

[44] Tulsawani R Kelly LS Fatma N Chhunchha B Kubo E Kumar A Neuroprotective effect of peroxiredoxin 6 against hypoxia-induced retinal ganglion cell damage. BMC Neurosci (2010) 11:125. 10.1186/1471-2202-11-125 20923568 PMC2964733

[45] DeFazio MV Rammo RA Robles JR Bramlett HM Dietrich WD Bullock MR. The potential utility of blood-derived biochemical markers as indicators of early clinical trends following severe traumatic brain injury. World Neurosurg (2014) 81:151–8. 10.1016/j.wneu.2013.01.015 23313262 PMC4974934

[46] Berger RPR Dulani TT Adelson PDP Leventhal JMJ Richichi RR Kochanek PMP. Identification of inflicted traumatic brain injury in well-appearing infants using serum and cerebrospinal markers: a possible screening tool. Pediatrics (2006) 117:325–32. 10.1542/peds.2005-0711 16452350

[47] Leclerc E Fritz G Vetter SW Heizmann CW. Binding of S100 proteins to RAGE: an update. Biochim Biophys Acta (2009) 1793:993–1007. 10.1016/j.bbamcr.2008.11.016 19121341

[48] Shanmugam N Kim YS Lanting L Natarajan R. Regulation of cyclooxygenase-2 expression in monocytes by ligation of the receptor for advanced glycation end products. J Biol Chem (2003) 278:34834–44. 10.1074/jbc.M302828200 12837757

[49] Lu J Goh SJ Tng PYL Deng YY Ling E-A Moochhala S. Systemic inflammatory response following acute traumatic brain injury. Front Biosci (Landmark Ed) (2009) 14:3795–813. 10.2741/3489 19273311

